# Periodontal Health among Individuals with Intellectual Disabilities Living in a Saudi Institutional Rehabilitation Centre

**DOI:** 10.3390/healthcare12090891

**Published:** 2024-04-25

**Authors:** Abdullah Ali H. Alzahrani

**Affiliations:** Dental Health Department, Faculty of Applied Medical Sciences, Al-Baha University, Al-Baha 65731, Saudi Arabia; aahalzahrani@bu.edu.sa; Tel.: +966-177257700 (ext. 14899)

**Keywords:** dental public health, disability, oral health, mental health, periodontal health, rehabilitation centre, special needs dentistry

## Abstract

The aim of this study was to explore periodontal health among intellectually disabled individuals living in an institutional rehabilitation centre in the Al-Baha Region of Saudi Arabia. A cross-sectional study was conducted from November 2023 to January 2024. Clinical oral examinations were carried out using the World Health Organization criteria for bleeding sites and the presence of periodontal pockets. Simplified oral hygiene and community periodontal indices were employed to evaluate participants’ oral health. A total of 89 participants, comprising both males and females with intellectual disabilities, were included in this study. Gender, severity of intellectual disability, type of additional physical disability, tooth brushing habits and oral hygiene status were significantly associated with the presence of periodontal disease. Additionally, poor oral hygiene, not brushing teeth and periodontal pockets of 4–5 mm and 6 mm or more were significantly more prevalent among individuals with severe and moderate intellectual disabilities (*p* = 0.001, *p* = 0.001, *p* = 0.001 and *p* = 0.001, respectively). The prevalence of periodontal disease among the studied population was 54%. The odds of having periodontal disease were significantly higher in the severe intellectual disability group compared to the mild intellectual disability group (OR = 2.328, 95% CI = 1.430–3.631, *p* = 0.03). It was also found that intellectually disabled participants with additional physical disabilities suffered more from periodontal disease than those without additional physical disabilities (OR = 0.971, 95% CI = 0.235–4.023, *p* = 0.025). Overall, individuals with intellectual disabilities had a significant demand for periodontal care. This study highlighted the need for more organised preventive programmes for individuals with intellectual disabilities. Dentists should be vigilant about improving periodontal health, focus on preventive programmes and provide comprehensive dental care with an emphasis on periodic recall and monitoring.

## 1. Introduction

The term disability refers to an individual’s impaired functioning, mental illness (including physical, sensory, cognitive and intellectual impairments) and different types of chronic diseases [1]. Impairment of the body or mind makes it more challenging for disabled individuals to carry out certain activities and interact with the world around them [2]. This condition is distinguished by major deficiencies in both intellectual functioning and adaptive behaviours, including conceptual, social and practical abilities [3]. Three key characteristics are prevalent among disabled individuals: considerable impairment of intelligence, a subsequent significant decline in adaptive behaviour/social functioning, and the onset of their condition before the age of 18 years, which then remains throughout life [4].

Oral health is an essential component of overall health and well-being. An individual’s oral health can be affected by a variety of conditions, including intellectual disabilities, developmental or physical disabilities, cerebral palsy, craniofacial anomalies and epilepsy. Oral health issues, which are common in individuals with disabilities, may occur due to individuals’ actual impairments, other medical illnesses, social circumstances, use of drugs or lack of accessibility to dental care, as well as due to their parents’ disregard for oral health [5]. This highlights the importance of assessing the oral health of this disabled group of individuals and developing appropriate healthcare and healthcare interventions to improve their health and well-being.

An individual’s capacity to obtain sufficient dental care can be hampered by a decline in mental or physical health, decreased vision or limited dexterity [6,7]. Poor oral hygiene practice, combined with inadequate dental health coverage and a lack of regular dental check-ups and restorative and/or surgical treatment in the dental office, increases the prevalence of poor oral hygiene and health, compromised periodontal health and dental caries [8]. Moreover, staying at home and indulging in cariogenic snacks and other eating habits may put disabled individuals at greater risk of dental caries. The necessity of dental care and the needs of chronically disabled individuals have frequently been overlooked by health planners and policy makers. This is mainly because these individuals may not understand the significance of and need for preventive dental hygiene and the consequences of poor oral health for their mouths and bodies [9,10,11,12,13,14,15,16,17,18,19]. Furthermore, the importance of reducing oral health inequalities worldwide among individuals with disabilities has been well reported, and improving accessibility to dental care provision has been emphasised [20]. However, numerous challenges and barriers for individuals with disabilities have been reported, including physical, behavioural and financial challenges. Most importantly, it is important to build constructive communication. The need for cooperation between patients, parents, carers/caregivers and practitioners to overcome these challenges has been emphasised [21,22,23].

Periodontitis is a disease caused by infection of the gum tissues. It has certain common symptoms, such as bad breath or taste, painful chewing and bleeding gums. Without periodontal care and therapy, bacteria grow in the mouth, which may cause pain, redness, swelling and tooth loss. Periodontitis has a negative impact on oral connective tissues around the teeth, causing major oral health issues, such as occlusal dysfunction and tooth loss [24]. Research has associated periodontitis with approximately 30–35% of all tooth extractions [25]. It is considered not only as a dental public health concern worldwide but also as a chronic inflammatory illness that has been linked to a variety of health issues, including heart disease, preterm birth, cancer, diabetes, rheumatoid arthritis, osteoporosis and chronic depression [26,27,28,29]. Moreover, worse quality-of-life outcomes have been reported among individuals with periodontitis compared to those who are periodontally healthy [30]. The complexity of periodontitis results from its association with several potential causative factors, including environmental factors, genetic and epigenetic aspects and different uses of medications [25]. Other research found that periodontitis was related to various risk factors, including poor oral hygiene, psychological stress, lower socioeconomic status, smoking, depression and increased age [31,32,33].

Evidence has revealed that the prevalence of periodontitis among Saudi adults is significantly higher than among other populations [34]. Most of the periodontitis research in Saudi Arabia seems to focus on investigating periodontitis among high-risk groups of patients, such as those with obesity or diabetes; however, researchers have not frequently studied the prevalence rate of periodontitis [34,35]. The Al-Baha region is one of 13 regions of Saudi Arabia. It is located in the southwestern part of the Kingdom. With a large population of approximately 500,000 people, it comprises several main cities, including Almandaq, Al-Baha, Buljurashi, Alatawelah and Alaqiq.

Exploring the prevalence of periodontal disease among disabled individuals is critical for developing effective prevention and treatment strategies that may advance the oral health of those individuals with disabilities. Due to the lack of existing evidence, this study is the first of its kind to explore periodontal disease among individuals with intellectual disabilities in Saudi Arabia. Therefore, the outcomes of this study may be used as baseline data for future research, which highlights the importance of this study. The aim of this study was to explore periodontal health among individuals with intellectual disabilities living in an institutional rehabilitation centre in the Al-Baha region of Saudi Arabia

## 2. Materials and Methods

### 2.1. Participants and Setting

The present cross-sectional study was conducted among participants residing in a rehabilitation centre in the Al-Baha region of Saudi Arabia from November 2023 to January 2024. The target population comprised intellectually disabled individuals aged 18 years and older of both genders. Data regarding severely disabled participants were obtained from carers/caregivers who were supervising these participants’ conditions. Data on their sociodemographic characteristics were collected in a specially designed format. The clinical diagnosis of the level of intellectual disability was obtained from participants’ medical records. The level was described as mild, moderate or severe in accordance with the Saudi Ministry of Health Guidelines and the International Classification of Diseases Version 10 [36]. Every individual with a disability was examined and assessed by a designated and qualified physician. The results of the assessments were registered in the patients’ medical records.

The rehabilitation centre had a total of 152 residents with disabilities. After applying the inclusion and exclusion criteria, 89 residents were identified as eligible for inclusion in the study. The inclusion criteria were as follows: first, participants were Saudi adults with intellectual disabilities; second, they were non-smokers; and third, they had at least 14 teeth or more at the time of the periodontal examination to guarantee meaningful assessment of their periodontal status [37]. The exclusion criteria were as follows: first, participants were under 18 years old; second, they were incapable of cooperating with oral examinations.

### 2.2. Clinical Oral Examination

The Saudi Ministry of Health Protocol was implemented. All clinical oral examinations were conducted in the rehabilitation centre. Clinical oral examinations based on World Health Organization criteria were carried out by two dental examiners in the participants’ rooms in the rehabilitation centre, with the aid of a mouth mirror, tweezers, a dental explorer, gauze, cotton, a face mask, gloves and a community periodontal index probe under adequate light [38]. The inter-examiner variability was tested, and the weighted kappa statistic was 0.86, indicating high agreement and consistency between the results of the two assessors. The clinical assessment of bleeding sites and the presence of pockets was conducted using the World Health Organization criteria [38], the simplified oral hygiene index (Greene and Vermillion) and the community periodontal index [39]. The modified version of the orthodontic treatment need index was used to evaluate malocclusion among participants [40].

Probing depth and attachment loss at six sites (buccal, distobuccal, mesiobuccal, lingual, distolingual and mesiolingual) on all teeth with the exception of the third molars were assessed during the clinical oral examinations of all participants included in the study. The Centers for Disease Control and Prevention classification from the Health and Nutrition Examination Survey and the American Academy of Periodontology Working Group classification were employed to group the periodontal cases into severe or moderate periodontitis. Severe periodontitis was identified once one or more interproximal site with a probing depth of ≥5 mm and two or more interproximal sites with attachment loss of ≥6 mm (not on the same tooth) were found. Moderate periodontitis was identified once two or more interproximal sites with a probing depth of ≥5 mm (not on the same tooth) or two or more interproximal sites with attachment loss ≥4 mm (not on the same tooth) were found [41,42].

### 2.3. Statistical Analysis

The Statistical Package for the Social Sciences Version 20.0 (IBM, Armonk, NY, USA) was used. A value of *p* < 0.05 was considered to indicate statistical significance. Frequency distribution was used for the descriptive analysis. A chi-squared test was conducted for the categorical variables. Logistic regression was also run to identify predictors of periodontal disease.

## 3. Results

A total of 89 participants, comprising both males and females with intellectual disabilities, were included in this study. The mean age of the study sample was 30.11 ± 4.39 years, while the mean duration of residence in the facility was 26.49 ± 4.66 years. Table 1 describes the sociodemographic characteristics of the participants.

There was a difference in the distribution of participants according to the severity of intellectual disability: mild (n = 39, 43.8%), moderate (n = 26, 29.2%) or severe (n = 24, 27%). Approximately 45% of participants had malocclusion, and all were on medication. Furthermore, the findings of this study revealed that gender, intellectual disability, type of additional disability, tooth brushing habits and oral hygiene status were significantly associated with the presence of periodontal disease. However, age group, malocclusion and comorbidity were not significantly associated with the presence of periodontal disease. Table 2 illustrates the association between participants’ demographic characteristics and periodontal status.

Interestingly, 100% of the study participants with moderate intellectual disabilities did not brush their teeth. By contrast, 54.2% of the severely disabled participants obtained assistance during brushing their teeth from carers/caregivers. This reflected how these severely disabled individuals were in a special care wing at the rehabilitation centre and received special support to maintain good oral hygiene due to their severe conditions. Moreover, the results showed that poor oral hygiene, not brushing teeth and having periodontal pockets of 4–5 mm or 6 mm or more were significantly prevalent among individuals with severe and moderate intellectual disabilities (*p* = 0.001, *p* = 0.001, *p* = 0.001 and *p* = 0.001, respectively). However, bleeding on probing was not significantly associated with the severity of intellectual disability. Table 3 describes the periodontal status of participants according to the severity of their intellectual disabilities.

Logistic regression was conducted to predict the presence of periodontal disease. The odds of having periodontal disease were significantly higher in the severe intellectual disability group compared to the mild intellectual disability group (OR = 2.328, 95% CI = 1.430–3.631, *p* = 0.03). Likewise, participants who did not brush their teeth were more exposed to periodontal disease than those who brushed (OR = 0.661, 95% CI = 0.073–1.597, *p* = 0.001). It was also found that intellectually disabled participants with additional physical disabilities suffered more from periodontal disease than those without additional physical disabilities (OR = 0.971, 95% CI = 0.235–4.023, *p* = 0.025). Moreover, individuals with poor oral hygiene status were more exposed to periodontal disease than those with good oral hygiene status (OR = 2.214, 95% CI = 1.397–3.915, *p* = 0.001). However, gender was not significantly associated with periodontal disease among the studied population. From these findings, it is clear that the severity of intellectual disability, the type of additional disability, tooth brushing practice and oral hygiene status were associated with the presence of periodontal disease. Table 4 provides details of the logistic regression analysis of the association between the independent variables and the presence of periodontal disease.

## 4. Discussion

One of the main purposes of the concept of dental public health is to support society-based illness prevention and the surveillance of oral health. Yet, there is insufficient evidence in the literature regarding the prevalence of periodontal disease among intellectually disabled adults in Saudi Arabia, especially those living in the Al-Baha region. Exploring this territory of research may not only open the horizon towards investigating the prevalence and severity of the disease among this group of people, but it may also help in developing appropriate preventive programmes and strategies for this group of individuals, fulfilling their dental treatment needs and improving their quality of life. Thus, the aim of this study was to explore periodontal health among individuals with intellectual disabilities living in an institutional rehabilitation centre in the Al-Baha region of Saudi Arabia.

The results of this study showed that the majority of participants with intellectual disabilities had periodontal changes (54%). Interestingly, among Saudi adults without disabilities, the prevalence of periodontal disease was almost the same (52%) as in our study [43]. By contrast, Tefera et al. (2022) reported that 71.3% of students living with disabilities had periodontal changes [9]. This difference in the prevalence and severity of periodontal disease can be attributed to the higher mean age in the present study. Students aged 18–30 years had a higher risk of having periodontal disease than students younger than age 18 [9]. This difference might also reflect the use of different methods to diagnose periodontal disease.

Most participants with intellectual disabilities in this study had a periodontal pocket depth of ≥4 mm (57.3%). Similar findings relating to the prevalence of a periodontal pocket depth of ≥4 mm were reported for individuals with other disabilities (61%, 49.6% and 96%) [44,45,46]. The variations in the prevalence rates for periodontal pocket depth might be due to underlying congenital or developmental anomalies, as well as the inability to receive adequate personal and professional care to maintain oral health. However, a minority of participants in this study had bleeding on probing (30.3%), which was consistent with previous research conducted among disabled individuals [9,47].

A statistically significant association between oral health status and periodontal disease was observed in the current study. In this respect, participants with poor oral health status were at high risk of having periodontal disease compared to participants with good oral health status (OR = 2.214, 95% CI = 1.397–3.915, *p* = 0.001). This was consistent with other research carried out among disabled individuals [9,48]. Furthermore, a systematic review and meta-analysis found that participants with poor oral hygiene status were at a 5.01 times greater risk of having periodontal disease than those with good oral hygiene status (OR = 5.01, 95% CI = 3.40–7.39) [49,50,51]. The poor oral health status of the disabled population might be due to their actual disabilities, social factors and medications. Thus, dental practitioners may have to pay attention to disabled groups of individuals to maintain good oral health by arranging regular dental visits and facilitating accessibility to dental care.

A significant association was found between tooth brushing habits and the severity of periodontal disease among intellectually disabled participants in this study. Similarly, Sermsuti-anuwat et al. (2019) reported an 8.25-fold risk of having periodontal disease among those who had poor tooth brushing habits compared to those who brushed their teeth frequently [46]. However, in contrast to the findings of the present study, Tefera et al. (2022) revealed no association between tooth brushing and periodontal disease [9]. This might be due to the fact that the majority of this study’s participants did not have appropriate tooth brushing habits, and almost none of them received carer/caregiver assistance while brushing their teeth.

The findings of the present study support existing evidence in the dental literature that emphasises the complexity of dental treatment needs, particularly periodontal needs, among intellectually disabled individuals [52]. Several variations have been reported in the literature that may be associated with the diversity of the prevalence of periodontal disease and treatment needs among intellectually disabled individuals. For example, the study design, the variability in clinical and demographic characteristics, the sample size, the inclusion and exclusion criteria and the appropriate conditions required for clinical examinations may together impact the findings of the research [53]. These variations may limit the generalisability and comparison of the present study to other research that has been conducted among intellectually disabled individuals. However, it is important to highlight the prevalence of periodontal disease in the present study, as well as the obvious unmet treatment needs of the studied population.

The present study not only emphasises the urgent requirement for robust efforts to better recognise the risk factors and periodontal health status of intellectually disabled individuals in Saudi Arabia, but it also advocates the need to explore the determinants of oral health, particularly periodontal status, among this group of people. Research has clearly shown that dental restorative care and tooth extractions due to periodontal issues are commonly required by intellectually disabled individuals [54]. This signifies the key role of dental practitioners in terms of implementing educational activities to improve oral health, prevent disease and provide instruction on oral hygiene practices and a healthy diet to carers/caregivers who are accountable for the oral health of intellectually disabled people. Regarding the oral healthcare of patients with disabilities, collaborative and integrated models of care among dentists, dental health educators, general medical practitioners, social workers, local authorities and caregivers may play a key role in improving the health and well-being of this population. For example, the potential role of dental health educators in improving the oral health of disabled people, including through collaborations and good relations with other stakeholders, organisations and healthcare providers, has been well reported [55]. Moreover, integrated models of dental care programmes, including teledentistry and oral care from dental therapists, have been demonstrated to improve the oral health status of institutionalised residents with disabilities [56]. It was shown that by using dental health educators, hygienists and therapists in institutional rehabilitation centres, the plaque scores of disabled residents were improved, and referrals to dental practitioners for additional therapy were effectively facilitated [57,58].

It is critical to recognise the limitations that are inherent in this study. First, the use of a convenience sampling methodology is subject to bias and is representative only of the population being studied. However, our study minimised this limitation by employing homogenous convenience sampling comprising various genders, age groups, disability types, medication intake, oral hygiene and tooth brushing habits. The second limitation was that this study did not measure dietary habits, which do have an impact on periodontal disease and oral health. Nonetheless, this study provides insight into periodontal health among intellectually disabled individuals living in the Al-Baha region of Saudi Arabia. To verify the results of the present study, a larger longitudinal study across Saudi Arabia that evaluates the periodontal status of intellectually disabled patients appears to be highly required.

Future research directions may focus on exploring oral-health-related quality of life, dietary habits and their association with the oral health of intellectually disabled individuals. Treatment needs and dental pain severity might also be examined among this disabled group of individuals. Systematic reviews of periodontal status among intellectually disabled individuals worldwide and especially in Saudi Arabia are also advocated. Moreover, a longitudinal study to assess the prevalence and progression of periodontitis and its relationship with tooth loss among intellectually disabled individuals is recommended. This will offer solid evidence highlighting this important issue at a policy level. The determinants and risk factors related to periodontitis among this disabled group of patients might also be explored in detail to provide clear guidelines for developing prevention strategies for this disease. To improve the oral health of disabled individuals, more efforts might be focused on the prevention, early diagnosis and treatment of periodontitis among intellectually disabled individuals in Saudi Arabia and elsewhere.

## 5. Conclusions

The prevalence of periodontal disease among the studied population was 54%. The odds of having periodontal disease were significantly higher in the severe intellectual disability group compared to the mild intellectual disability group (OR = 2.328, 95% CI = 1.430–3.631, *p* = 0.03). Intellectually disabled participants with additional physical disabilities suffered more from periodontal disease than those without additional physical disabilities (OR = 0.971, 95% CI = 0.235–4.023, *p* = 0.025). The dental workforce could develop plans to fulfil the dental treatment needs of the studied population. This study highlights the need for more organised preventive strategies and programmes for intellectually disabled adults. Interventions may also be developed that target improving the oral health of the studied population. Dentists should be vigilant about improving periodontal health, focusing on preventive programmes, educating parents and providing comprehensive dental care. Emphasis should also be placed on the health education and periodic recall and monitoring of intellectually disabled individuals.

## Figures and Tables

**Table 1:** Demographic characteristics of participants.

Variable		n (%)
Age Group	18–25 years	1 (1.1)
	26–30 years	58 (65.2)
	31–35 years	21 (23.6)
	36 years and older	9 (10.1)
Gender	Female	37 (41.6)
	Male	52 (58.4)
Intellectual Disability	Mild	39 (43.8)
	Moderate	26 (29.2)
	Severe	24 (27)
Type of Additional Disability	None	45 (50.6)
	Hearing	1 (1.1)
	Physical	39 (43.8)
	Visual	4 (4.5)
Malocclusion	Yes	40 (44.9)
	No	49 (55.1)
Medication	Yes	89 (100)
	No	0
Comorbidity	Yes	59 (66.3)
	No	30 (33.7)
Tooth Brushing	Yes	22 (24.7)
	No	67 (75.3)
Oral Hygiene Status	Good	13 (14.6)
	Fair	32 (36.4)
	Poor	44 (49.4)

**Table 2:** Association between participants’ demographic characteristics and periodontal status.

Variable		Presence of Periodontal Disease n (%)	Absence of Periodontal Disease n (%)	Chi-Squared Test	*p*-Value
Age Group	18–25 years	1 (100)	0 (0)	1.576	0.665
	26–30 years	30 (51.7)	28 (48.3)		
	31–35 years	11 (52.4)	10 (47.6)		
	36 years and older	6 (66.7)	3 (33.3)		
	Total	48 (54)	41 (46)		
Gender	Female	25 (67.6)	12 (32.4)	4.739	0.024 *
	Male	23 (44.2)	29 (55.8)		
	Total	48 (54)	41 (46)		
Intellectual Disability	Mild	31 (79.5)	8 (19.5)	39.721	0.001 *
	Moderate	17 (65.4)	9 (34.6)		
	Severe	0 (0)	24 (100)		
	Total	48 (54)	41 (46)		
Type of Additional Disability	None	33 (73.3)	12 (26.7)	16.428	0.001 *
	Hearing	0 (0)	1 (100)		
	Physical	15 (38.5)	24 (61.5)		
	Visual	0 (0)	4 (100)		
	Total	48 (54)	41 (46)		
Malocclusion	Yes	20 (50)	20 (50)	0.452	0.323
	No	28 (57.1)	21 (48.9)		
	Total	48 (54)	41 (46)		
Medication	Yes	48 (54)	41 (46)	NA	NA
	No	0 (0)	0 (0)		
	Total	48 (54)	41 (46)		
Comorbidity	Yes	33 (56)	26 (44)	0.282	0.379
	No	15 (50)	15 (50)		
	Total	48 (54)	41 (46)		
Tooth Brushing	Yes	5 (22.7)	17 (77.3)	11.454	0.001 *
	No	43 (64.2)	24 (35.8)		
	Total	48 (54)	41 (46)		
Oral Hygiene Status	Good	10 (77)	3 (23)	13.884	0.001 *
	Fair	23 (71.8)	9 (28.2)		
	Poor	15 (34.1)	29 (65.9)		
	Total	48 (54)	41 (46)		

Chi-squared test; * *p* = statistically significant; NA = not available.

**Table 3:** Periodontal status among participants according to severity of intellectual disability.

Variable		Mild (n = 39)	Moderate (n = 26)	Severe (n = 24)	Total (n = 89)	Chi-Squared Test	*p*-Value
Bleeding on Probing	Yes	15 (38.5)	8 (30.7)	4 (16.7)	27 (30.3)	3.343	0.188
	No	24 (61.5)	18 (69.3)	20 (83.3)	62 (69.7)		
Periodontal Pocket of 4–5 mm	Yes	18 (46.2)	14 (53.8)	24 (100)	51 (57.3)	23.834	0.001 *
	No	21 (53.8)	12 (46.2)	0 (0)	38 (42.7)		
Periodontal Pocket of 6 mm or more	Yes	27 (69.2)	11 (42.3)	0 (0)	48 (53.9)	29.107	0.001 *
	No	12 (30.8)	15 (57.7)	24 (100)	41 (46.1)		
Tooth Brushing	Yes	9 (23.1)	0 (0)	13 (54.2)	22 (24.7)	19.778	0.001 *
	No	30 (76.9)	26 (100)	11 (43.8)	67 (75.3)		
Oral Hygiene Status	Good	13 (33.3)	0 (0)	0 (0)	13 (14.6)	48.724	0.001 *
	Fair	19 (48.7)	13 (50)	0 (0)	32 (36.4)		
	Poor	7 (18)	13 (50)	24 (100)	44 (49.4)		

Chi-squared test; * *p* = statistically significant.

**Table 4:** Logistic regression analysis of the association between the independent variables and periodontal disease.

Variable		*p*-Value	Odds Ratio	95% CI: Upper Limit	95% CI: Lower Limit
Gender	Female *				
	Male	0.372	1.564	0.778	2.330
Intellectual Disability	Mild *				
	Moderate	0.10	1.272	0.903	2.431
	Severe	0.03 **	2.328	1.430	3.631
Type of Additional Disability	None *				
	Hearing	0.968	1.793	1.012	2.115
	Physical	0.025 **	0.971	0.235	4.023
	Visual	0.651	1.802	1.116	2.693
Tooth Brushing	Yes *				
	No	0.001 **	0.661	0.073	1.597
Oral Hygiene Status	Good *				
	Fair	0.354	1.637	1.042	2.539
	Poor	0.001 **	2.214	1.397	3.915

Logistic regression analysis; * reference; ** *p* = statistically significant.

## Data Availability

The data are available for research purposes upon request (please email Dr Alzahrani: aahalzahrani@bu.edu.sa).
